# Pathologic Complete Response to Neoadjuvant Chemotherapy and Pembrolizumab in Postpartum High-Risk Basal-Type Breast Cancer

**DOI:** 10.7759/cureus.62338

**Published:** 2024-06-13

**Authors:** Heng Jiang, Sara Cartwright, David G Wagner, Jairam Krishnamurthy, Juan A Santamaria-Barria

**Affiliations:** 1 Department of Medicine, Westchester Medical Center, Valhalla, USA; 2 Department of Surgery, University of Nebraska Medical Center, Omaha, USA; 3 Department of Pathology and Microbiology, University of Nebraska Medical Center, Omaha, USA; 4 Department of Hematology/Oncology, University of Nebraska Medical Center, Omaha, USA; 5 Department of Surgery, Division of Surgical Oncology, University of Nebraska Medical Center, Omaha, USA

**Keywords:** triple-negative breast carcinoma, cancer immunotherapy, pregnancy-associated breast cancer, immunotherapy in er+metastatic breast cancer, breast cancer outcomes

## Abstract

Neoadjuvant chemoimmunotherapy with pembrolizumab now defines the standard of care for early high-risk triple-negative breast cancer (TNBC). However, the role of pembrolizumab in neoadjuvant therapy (NAT) for estrogen receptor-positive (ER+) breast cancer remains uncertain. A 39-year-old G2P2 female discovered a palpable mass in the right breast while breastfeeding her 7-month-old child, leading to the diagnosis of a high-grade ER+ (80% moderate staining), human epidermal growth factor receptor 2-negative (ErbB2-) invasive ductal carcinoma with axillary nodal involvement. Gene expression profiling with the MammaPrint 70-gene signature and BluePrint 80-gene signature revealed a tumor with high-risk, basal-type biology.

The multidisciplinary breast cancer team recommended NAT with pembrolizumab, carboplatin, paclitaxel, doxorubicin, and cyclophosphamide. Within six weeks, the patient exhibited a remarkable response, with no palpable mass or lymph node, and post-treatment examinations confirmed a complete clinical and radiologic response. The patient underwent lumpectomy and sentinel lymph node biopsy, revealing a pathological complete response with minimal ductal carcinoma in situ and negative axillary nodes. Adjuvant radiation therapy was administered, and the patient completed adjuvant pembrolizumab, currently showing no evidence of recurrence.

This case underscores the potential benefits of neoadjuvant chemoimmunotherapy for patients with ER+ErbB2- high-risk, basal-type breast cancer. The use of immunotherapy in patients with pregnancy-associated breast cancer remains to be further investigated.

## Introduction

Estrogen receptor-positive, ErbB2 (formerly HER2/neu)-negative (ER+ErbB2-) breast cancer is typically endocrine therapy sensitive and poorly responsive to systemic chemotherapy. However, a small subset of ER+ErbB2- breast cancers are chemosensitive and achieve pathological complete responses (pCR) to chemotherapy-based neoadjuvant systemic therapy (NAT) [1]. In high-risk triple-negative breast cancer (TNBC), the combination of neoadjuvant immunotherapy and chemotherapy is now the standard of care [2]. The KEYNOTE-522 trial reported improvement in pCR and event-free survival among high-risk TNBC patients receiving NAT with pembrolizumab compared to placebo [3]. For ER+ErbB2- disease, the use of NAT consists of chemotherapy, which does not result in significant responses [1]. The Neoadjuvant Breast Registry Symphony Trial (NBRST) showed that ER+ErbB2- patients who qualified for NAT with MammaPrint high-risk, BluePrint basal-type responded to NAT similarly to TNBC [4, 5]. The KEYNOTE-756 phase III randomized trial (NCT03725059) is investigating the addition of pembrolizumab to NAT and standard adjuvant endocrine therapy in high-grade ER+ErbB2- breast cancer, but no gene expression profiling is being included in this trial [6]. Thus, we present a case of pCR to pembrolizumab and NAT in a postpartum ER+ErbB2- breast cancer patient with MammaPrint high-risk and BluePrint basal-type gene expression molecular profiling.

## Case presentation

**Figure 1:**
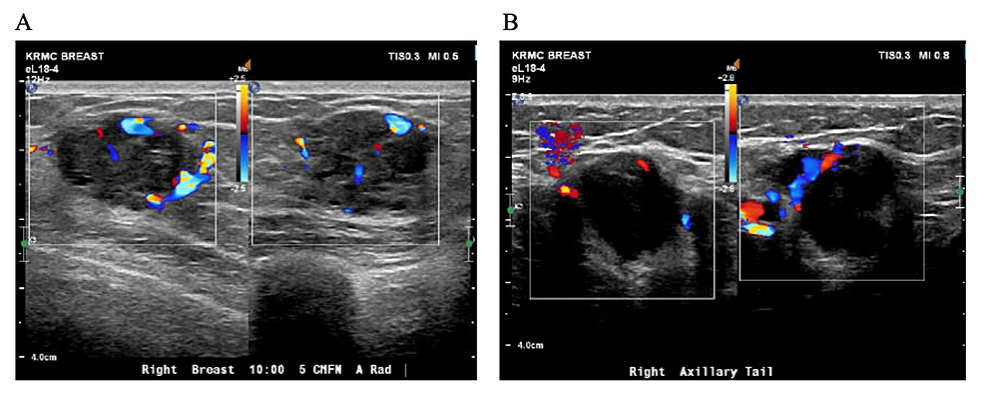
Right breast ultrasound. A right breast mass at 10:00, 5 cm from the nipple, measuring 2.4 cm (A) and a right axillary tail abnormal lymph node measuring 2.3 cm (B).

A 39-year-old G2P2 female noticed a palpable right breast mass while breastfeeding her seven-month-old baby in November 2022. She otherwise had no other symptoms, and the pregnancy and delivery were uncomplicated. The patient had undergone several rounds of in vitro fertilization. Initially, the mass was attributed to a clogged duct by an outside facility, and the patient was reassured. However, the mass continued to grow according to the patient, prompting a right breast ultrasound, which demonstrated two irregular hypoechoic masses: one located 5 cm from the nipple at the 10 o'clock position measuring up to 2.4 cm, and another abnormal axillary tail lymph node measuring 2.3 cm (Figure 1).

**Figure 2:**
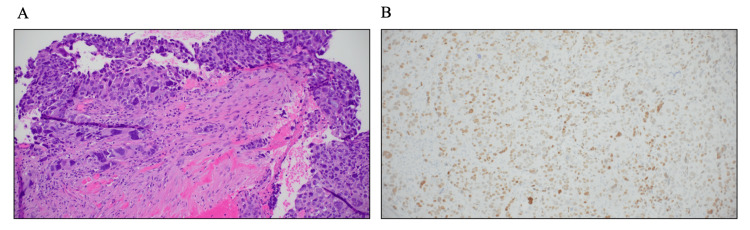
Right breast core needle biopsy pathology. Hematoxylin & eosin showing Nottingham grade 3/3 invasive ductal carcinoma with significant nuclear atypia, pleomorphism, and large giant cells with anaplastic type morphology (A; 20x), with estrogen receptor positive at 80% with moderate staining intensity (B; 20x).

On physical examination, there was a large 5 cm area of palpable mass and fullness in the upper outer right breast, which was engorged from breastfeeding, and a single palpable mobile 3 cm axillary tail nodule. Core needle biopsies were obtained, and the pathology of the breast mass showed Nottingham grade 3/3 invasive ductal carcinoma with significant nuclear atypia, pleomorphism, and large giant cells with anaplastic-type morphology, with estrogen receptor positive at 80% with moderate staining intensity (Figure 2), progesterone receptor negative at 0% staining intensity, ErbB2 negative at 0+, and a high Ki-67 of >90%. The right axillary lymph node showed metastatic adenocarcinoma of breast origin with the same molecular profile but weaker estrogen receptor expression at 40% with moderate staining intensity and both progesterone receptor and ErbB2 negative at 0% staining.

**Figure 3:**
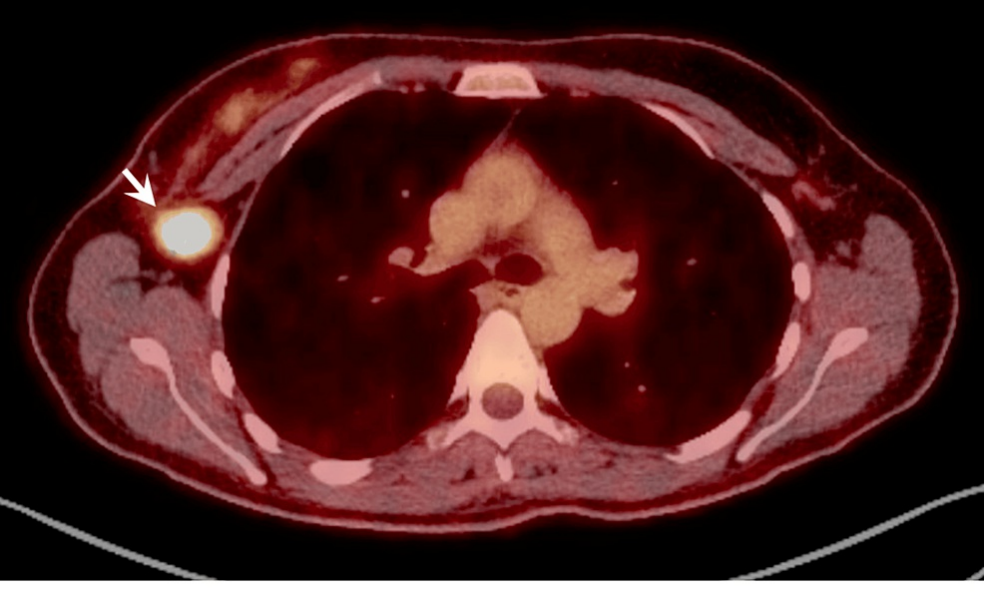
Right breast PET/CT scan. A single right axillary node measuring 2.2 cm with SUV-max of 11 (white arrow).

A breast magnetic resonance imaging (MRI) showed the breast mass measured 5.8 cm with a single enlarged axillary lymph node of 2.1 cm. A positron emission tomography-computed tomography (PET/CT) showed a right 7.9 cm area of abnormal breast avidity with a maximum standardized uptake value (SUV-max) of 7, a single right axillary node measuring 2.2 cm with an SUV-max of 11 (Figure 3), and no distant metastatic disease. The patient was anatomically staged as IIIA (cT3N1M0) and prognostically as IIIB (cT3N1M0 high-grade 3/3 ER+PR-ErbB2-).

The patient was discussed at our multidisciplinary breast cancer tumor board, which included specialists from surgical oncology, medical oncology, radiation oncology, and pathology. The final recommendation was NAT as the first treatment strategy, followed by breast-conserving surgery, limited axillary surgery if a complete nodal clinical response was obtained (ycN0), and adjuvant radiation therapy. The patient underwent multi-panel genetic testing, which was negative. When choosing the NAT regimen, the multidisciplinary team’s consensus was that the tumor biology was more representative of high-risk, basal-type, triple-negative breast cancer. The weaker estrogen receptor staining in the metastatic axillary lymph node (40% moderate intensity) further supported the team’s sentiment. A 70-gene MammaPrint signature categorized the tumor as high-risk group (index of -1.0), and the 80-gene BluePrint signature showed the molecular subtype as basal-type.

Thus, the multidisciplinary team proposed the KEYNOTE-522 chemoimmunotherapy regimen of pembrolizumab combined with carboplatin, paclitaxel, doxorubicin, and cyclophosphamide. The patient was thoroughly informed about the trial inclusion criteria and results, and she consented to receiving this regimen. The patient received pembrolizumab (200 mg) every three weeks along with carboplatin (AUC of 5 every three weeks or 1.5 mg once weekly in the first 12 weeks) and paclitaxel (80 mg per square meter of body-surface area once weekly) followed by four cycles of doxorubicin (60 mg per square meter) and cyclophosphamide (600 mg per square meter).

After six weeks of treatment, the breast mass and lymph node were no longer palpable. While on therapy, she reported symptoms of intermittent dizziness, fatigue, and hot flashes. She otherwise tolerated chemoimmunotherapy well, but refused the last fourth cycle of doxorubicin and cyclophosphamide due to profound fatigue.

**Figure 4:**
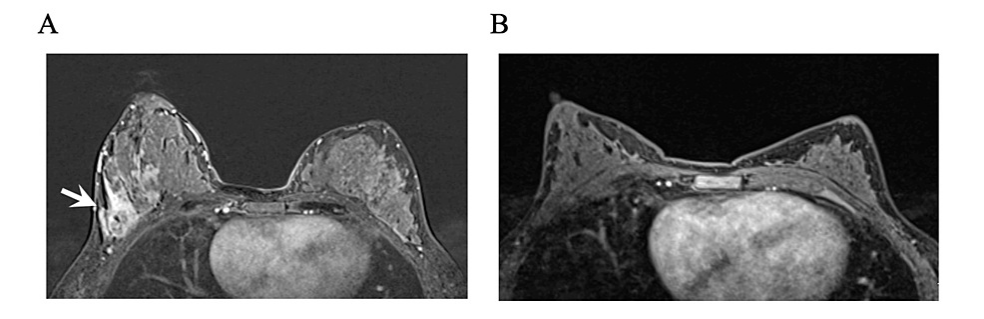
Bilateral breast MRI. Contrast-enhancing 5.8 cm mass in the upper outer quadrant of the right breast (white arrow) in the background of enlarged, enhancing, and increased fibroglandular breast tissue from breastfeeding (A), and complete radiological response in the right breast and axilla obtained post-treatment with no areas of mass or contrast enhancement present (B).

**Figure 5:**
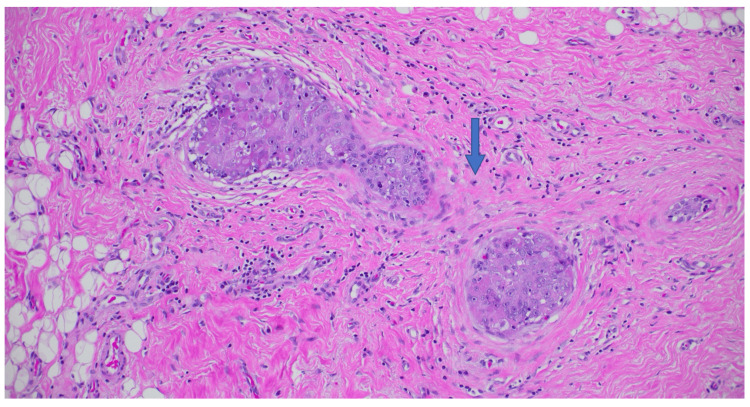
Right breast lumpectomy pathology. Hematoxylin & eosin pathology showing no residual invasive cancer post neoadjuvant therapy (pCR, ypTis ypN0(i-)) with a 0.2 cm focal high-grade ductal carcinoma in situ (blue arrow; 20x), with variable dense fibrosis, reactive stromal cells, histiocytes, and background fibrocystic-type changes.

Post-treatment exam and breast MRI suggested a complete response, ycT0 ycN0 (Figure 4). She then underwent breast-conserving right lumpectomy, removal of the axillary clipped node, and sentinel lymph node biopsy. Surgical pathology revealed pCR with a 0.2 cm focus of ductal carcinoma in situ (DCIS; Figure 5), and all eight axillary lymph nodes excised were free of carcinoma, pathological stage ypTis ypN0(i-) with a residual cancer burden index of 0 (RCB-0). The patient then completed adjuvant radiation to the right breast and regional nodes to 50 Gy in 25 fractions, followed by a boost of 10 Gy in five fractions to the lumpectomy scar bed. She finished adjuvant pembrolizumab and remained free of recurrence at the last follow-up.

Written informed consent was obtained from the patient, granting us permission to report deidentified information, including clinical, radiologic, and pathologic data. This study was approved by the Institutional Review Board at the University of Nebraska Medical Center.

## Discussion

The use of immunotherapy in breast cancer was initially evaluated in the metastatic setting and has been mostly successful in TNBC. Recently, studies have investigated its role in the neoadjuvant setting [7]. ER+ErbB2- breast cancers have a relatively low response to chemotherapy with an approximate pCR rate of 5%-15%, indicating a variable spectrum of tumor biology and chemotherapy responsiveness [8]. MammaPrint, a 70-gene expression assay that stratifies ER+ErbB2- breast cancers into low-risk (luminal A) and high-risk (luminal B), has been investigated in a phase III clinical trial (MINDACT TRIAL: NCT00433589), which showed promising results in selecting patients who can avoid adjuvant chemotherapy [9]. BluePrint, an 80-gene expression assay, stratifies breast cancer into three distinct molecular subtypes: luminal, HER2, and basal [10]. Both assays have been shown to identify patients who are more likely to respond well to chemotherapy [4, 11].

Our patient was classified as MammaPrint high-risk with the lowest index possible (-1.0) and BluePrint basal-type, a tumor biology that resembles TNBC and most likely is not dependent on estrogen receptor signaling [12]. This tumor biology supports the possibility that it is likely to respond well to chemotherapy and possibly immunotherapy [4, 13]. Therefore, we treated this patient with the KEYNOTE-522 chemoimmunotherapy regimen of pembrolizumab and chemotherapy. After successful completion of this regimen, the patient sustained pCR with a small focus of DCIS and no nodal metastases, suggesting the potential benefit of neoadjuvant chemoimmunotherapy in treating a subset of ER+ErbB2- patients with high-risk, basal-type breast cancer.

Though no definite recommendations for chemoimmunotherapy in ER+ErbB2- patients have been made, there are several trials currently addressing this topic (Table 1). The KEYNOTE-756 trial (NCT03725059) completed accrual and is currently investigating the addition of pembrolizumab to NAT and adjuvant endocrine therapy in high-grade (3/3) ER+ErbB2- breast cancer patients. The first update from this trial showed that the pCR co-primary endpoint was met and the event-free survival co-primary endpoint is pending [6]. SWOG has recently initiated a phase III randomized trial for ER+ErbB2- patients with an ultrahigh MammaPrint index (MP2) to receive neoadjuvant immunotherapy as well (NCT06058377) [14]. Caution is required when relying solely on pCR as a surrogate endpoint in clinical trials since this may not fully reflect long-term outcomes in the real-world setting, especially in ER+ErbB2- breast cancer, which is known for late recurrences [15].

**Table 1:** Clinical trials studying neoadjuvant systemic therapy with immunotherapy in estrogen receptor-positive, ErbB2 receptor tyrosine kinase 2-negative breast cancer. Drugs in bold are the immunotherapy drugs being studied. *379 patients on pembrolizumab and durvalumab arms so far. ^#^Most frequent potentially immune-related adverse events during nivolumab were endocrinopathies (all grades 1–2), including hyperthyroidism (11.9%), hypothyroidism (14.3%), adrenal insufficiency (2.4%), and ACTH decrease (4.8%) [18]. pCR: pathological complete response; AC: adriamycin + cyclophosphamide; MP2: MammaPrint High 2; BP: BluePrint; HR+: hormone positive; BC: breast cancer; HER2: human epidermal growth factor 2; NACT: neoadjuvant chemotherapy; SBRT: stereotactic body radiation therapy.

Trial name	Registration	Phase	Drugs	Status	Endpoints	Number of subjects	pCR	Toxicity and adverse events
CheckMate7A8 [16]	NCT04075604	II	Nivolumab + palbociclib + anastrozole	Early termination: toxicity	pCR and event-free survival	21	1 patient (5% )	High incidence of grade 3/4 hepatotoxicity and treatment discontinuations
I-SPY 2 [17]	NCT01042379	II	Pembrolizumab + AC; durvalumab + olaparib + paclitaxel vs. AC	Ongoing	pCR and event-free survival	379^*^	55% vs 21%- MP2 and BP Basal signatures HR+/HER2- BC subset more likely to respond to neoadjuvant therapy	11%, immune-related grade 3 adverse events
GIADA [18]	NCT04659551	II	Epirubicin cyclophosphamide + nivolumab, triptorelin	Completed	pCR and event-free survival	43	7 (16.3%); 4/8 50% in PAM50 basal BC	42.9%, immune-related adverse events, mostly endocrinopathies^#^
Neo-CheckRay [19]	NCT03875573	II	NACT + SBRT +/− durvalumab +/− oleclumab	Ongoing	pCR and event-free survival	147		
MCC-15-11083 [20]	NCT02957968	II	Decitabine + pembrolizumab AC	Ongoing	pCR and event-free survival	46		
MK-3475/P-RAD [21]	NCT04443348	II	Pembrolizumab + radiation & NACT	Recruiting	pCR and overall survival	Estimated 120		
KEYNOTE-756 [22]	NCT03725059	III	Pembrolizumab + chemotherapy vs placebo + chemotherapy	Ongoing	pCR and event-free survival	Estimated 1240	pCR reported improvement with pembrolizumab (press release)	
MEDI4736 [23]	NCT03132467	I	Tremelimumab + durvalumab	Early termination: toxicity	pCR and overall survival	16	0% - likely secondary to early termination	37%, trial stopped early after 2/8 patients experienced G3 immune-related adverse events

The other interesting aspect of this case was that the cancer could be defined as postpartum breast cancer (PPBC), which usually occurs within six months to one year of delivery, but recent data suggest PPBC can occur up to 10 years after the last pregnancy [24, 25]. PPBC is associated with poorer survival and increased rates of metastasis compared to breast cancer diagnosed during pregnancy or in premenopausal, nulliparous women [24, 26]. The cancer in our patient demonstrated aggressive, high-grade features and nodal involvement. There are no current clinical guidelines or consensus to standardize the management of PPBC or pregnancy-associated breast cancer, and the effect of immunotherapy and NAT in such patients also remains unknown. With limited evidence available, pembrolizumab is not recommended during pregnancy, and breastfeeding should be discontinued during therapy and for four months after the last dose [27].

To our knowledge, this is the first case reporting a pCR to neoadjuvant chemoimmunotherapy in a PPBC patient with ER+ErbB2-, MammaPrint high-risk, and BluePrint basal-type breast cancer as a personalized neoadjuvant treatment strategy. This case suggests that some ER+ErbB2- basal-type breast cancer patients could benefit from neoadjuvant chemoimmunotherapy, and we eagerly await further evidence from KEYNOTE-756, CheckMate 7FL, and the MammaPrint-tailored S2206 randomized phase III trials. More research is needed on tumor biology and the best treatments for pregnancy-associated and PPBC patients. For tailored treatment plans, we should provide fertility counseling, preservation options, and genetic testing to young women with breast cancer. Additionally, offering onco-lactation resources and education is important for patients with postpartum and pregnancy-associated breast cancer.

## Conclusions

In conclusion, the presented case highlights the potential efficacy of neoadjuvant chemoimmunotherapy in treating high-risk, basal-type ER+ErbB2- breast cancer, particularly in achieving pathological complete response. It also emphasizes the increasing interest and necessity for tailored treatment approaches based on molecular profiling, such as MammaPrint and BluePrint assays. Furthermore, the unique aspect of postpartum breast cancer in this case underscores the importance of exploring effective novel treatment strategies for this subset of patients. Moving forward, further exploration of tumor biology and therapeutic interventions, particularly in the context of pregnancy-associated and postpartum breast cancer, is essential to enhance patient outcomes.
